# Franz Joseph Gall's non‐cortical faculties and their organs

**DOI:** 10.1002/jhbs.21994

**Published:** 2019-09-09

**Authors:** Paul Eling, Stanley Finger

**Affiliations:** ^1^ Department of Psychology, Donders Institute for Brain, Cognition and Behaviour Radboud University Nijmegen Nijmegen The Netherlands; ^2^ Department of Psychological and Brain Sciences Washington University Saint Louis Missouri

## Abstract

Franz Joseph Gall (1758–1828) is remembered for his claims that behavior results from a large number of independent mental faculties, and that these faculties are associated with cortical organs. Apart from the 26 faculties he localized in the cerebrum, he also recognized one faculty (reproductive drive) in the cerebellum. This picture, however, is based on Gall's presentations in his well‐known later works, his four volume *Anatomie et Physiologie.* These books reflect the outcomes of Gall's thinking. They were steered by the observations and feedback he received in Vienna and while presenting his theories in the German states and neighboring countries between 1805 and 1807. Examining his lists before what he published in Paris shows how his faculties were changing. Notably, and as shown here, he had previously included several faculties associated with brainstem structures, in addition to the cerebellum, which he would continue to associate with some reproductive behaviors.

## 1 INTRODUCTION

Franz Joseph Gall (1758–1828) was born in 1758 in the small, southern German town of Tiefenbronn and died in 1828 in Paris. He began studying medicine in Strasbourg and completed his medical studies in Vienna in 1785, where he began a lucrative private practice. Stimulated by the writings of philosopher Johan Gottfried von Herder (1744–1803) and his own interests in nature, he began to study differences in behavior and neuroanatomy among humans and other species, with the goal of linking together what he was seeing in decidedly natural ways.

In 1791, Gall published a medical book in which he rejected classical philosophical views and metaphysics. In his passages, he was already calling for a new and better scientific framework for understanding the mind, the brain, and behavior, though it lacked the specifics of the doctrine that would soon bring him fame (Gall, 1791).

Up to the 18th and even into the 19th century, philosophers and scientists, in general, had thought of the soul or mind as a special, typically nonmaterial entity associated with the physical body for perceiving, judging, and storing images. The idea that a reigning soul should be associated with the ventricles had been formulated in antiquity by Greek philosophers and anatomists. Citing the authority of Galen (ca. 130–200), who practiced medicine in Rome, the notion of “animal” (from *anima*, meaning soul) spirits and ventricular localization of higher functions (e.g., perception and cognition memory) became dogma (Smith et al., 2012).

Philosophers interested in the workings of the nervous system adhered to this general view, although some strove to fine‐tune the doctrine (e.g., the nature of the spiritous matter; which ventricle should be associated with each function or functions). Some scholars, for example, distinguished not three but four or five higher faculties, while others further pondered whether the spirits are immaterial or material (Manzoni, 1998). During the 17th century, influential French philosopher René Descartes (1596–1650) made a sharp distinction between the mind (which he equated with the human soul) and the physical body, and claimed that the former, or the *res cogitans* as he referred to it, should be regarded as an indivisible entity. Although Descartes had a following, other anatomists, following Thomas Willis (1621–1675), began to abandon the ventricles, arguing that, in one way or another, different parts of brain itself must be involved. Importantly, what endured was still very speculative, being tied to religious beliefs, especially metaphysical notions about invisible spirits and nature of the guiding soul (Smith et al., 2012).

Although he was primarily driven to understand more about the soul and God's kingdom, Swedish philosopher Emanuel Swedenborg (1688–1772) achieved a better understanding of the functional organization of the cerebral cortex during the mid‐eighteenth century (Gross, 2009). Citing clinical cases mostly described by others, as well as anatomical discoveries, Swedenborg astutely recognized that discrete regions of the cortex must be specialized for different functions. This insight pertained to the different senses, voluntary movements, and higher mental processes (which he associated with the front of the brain). In some ways, Swedenborg preceded Gall's views, but his ideas about the brain did not circulate and were little recognized during his lifetime, in part because he did not publish his most important findings, but also because of his visions and religious mysticism, which led him to abandon these studies while rewriting scripture. Gall, who was an avid reader, would mention Swedenborg's religious visions in his own books, although he seemed unaware of the Swede's earlier insights about cortical localization of function (see Finger & Eling, 2019).

Adhering to time‐honored theories and preconceived notions can be counted among the many reasons why physicians made so little progress in understanding brain organization and physiology before the 19th century. But there were also other factors, including the tools available and how investigators went about examining brains. Not only did these men lack the means to preserve brains adequately for later study, but dissections usually proceeded from the top downwards, making it very difficult to follow the fibrous pathways between various parts. Gall deserves credit for being one of the first anatomists to separate the gray from the white matter, and for following tracts up to the cerebral cortex. These achievements, combined with how he related humans to other animals on his Great Chain of Being (notably lacking metaphysical entities at the top) and what he was observing in patients with brain diseases and injuries, led him to his new framework for understanding the brain, the mind, and behavior.

During the mid‐1790s, Gall began to invite students, physicians, and a broad range of citizens to his stately house in Vienna, where he kept his growing skull and cast collections and gave public lectures. It was during this time that he started to formulate a list of mental faculties, each for a specific, practical, and independent behavioral trait, such as language, music, mathematics, each with its own memory. He was now maintaining that each fundamental faculty could be associated with a specific part of the brain, most of which could be revealed by studying skull morphology. In his later works, Gall would go on to describe not only the basic features of each of his chosen faculties, but also how he “discovered” (his word) most of them. But in 1798, he did no more than to present some of his methods and the principles of his new theoretical framework to the public in a letter to the Austrian censor that was published in a German periodical (Finger & Eling, 2019; Gall, 1798). He did not provide his nascent list of faculties in this publication.

By the start of the new century, opposition to his views was growing in conservative Vienna, where some people were maintaining he was preaching a materialistic doctrine, and with it, loss of free will. In 1802, Emperor Franz Joseph II (1768–1835), guided by his medical advisor, Andreas Joseph von Stifft (1760–1836), and feeling under pressure to maintain order, prohibited Gall from lecturing at home without official permission—something Gall recognized he was not about to receive. After a few years of being curtailed from presenting his doctrine to the Viennese public, Gall decided to make a scientific journey through German states. His objectives were to obtain more recognition for his revelations, to gather additional case studies, and to obtain needed feedback for the book or books he had already started to write. Leaving Vienna at this time also allowed him to visit his aging parents, who still resided in Tiefenbronn.

Gall's first lectures were presented in Berlin in 1805. Many more followed before what he had thought would be a tour of a few months took him to Paris in 1807, where he spent the rest of his life. His stops in various cities in what is now Germany, Denmark, the Netherlands, and Switzerland, were covered by local and foreign newspapermen. Importantly, some authors took extensive notes and published them, providing detailed lists of Gall's faculties of mind, which were very much in flux at this time.

What caught our attention when reading these early reports was that, before arriving in Paris, Gall was including several faculties not associated with the cerebral cortex (which we will henceforth refer to as his noncortical faculties) on his lists. These noncortical faculties were always listed before the reproductive faculty, the one faculty he associated with the cerebellar cortex (Eling & Finger, 2019), and his large number of cerebral cortical faculties, starting with the most primitive and working up to those he viewed as exclusively human. Gall's noncortical faculties would not, however, survive. They would be purged from the lists he would publish in Paris in his two sets of volumes.

It is important to note at this juncture that there is a large literature on Gall and his finalized doctrine, and that the individual items range from scholarly treatises to gross misrepresentations. Among the better works are the books and articles written by Ackerknkecht and Vallois (1953), Young (1970), Temkin (1947), Lesky (1970, 1979), Oehler‐Klein (1990), and Van Wyhe (2002). Nonetheless, even these explorations provide little or no information about how he settled on his individual faculties and, even more strikingly, what had been on his earlier lists.

With the latter foremost in mind, we reexamined various accounts of Gall's lectures spanning the years 1802 until 1806, trying to get a better sense of what he had been saying about what were destined to become his discarded noncortical faculties. Since Gall was not yet ready to give a full account of his faculties in a book or even an article, we could only rely on the second‐hand accounts coming from people who attended and took notes during his lectures in Vienna and while travelling. What we found and shall now present chronologically differs significantly from what Gall would later publish in his *Anatomie et Physiologie du Système Nerveux en Général, et du Cerveau en Particulier* (Gall & Spurzheim, 1810–19) and repeat in his less‐expensive follow‐up book, his *Sur les Fonctions du Cerveau et sur Celles de Chacune de ses Parties* (Gall, 1822–25).

Overlooked for too long, we believe this history merits more attention from those interested in the emergence of what is now called phrenology, the catchy term popularized by Gall's estranged assistant Johann Gaspar Spurzheim (1776–1832; Gall preferred “organology” and other terms). Although bits and pieces of what we shall now present appeared in our recent scientific biography of Gall (Finger & Eling, 2019), readers will find the present survey of Gall's long forgotten noncortical organs to be much more detailed.

## 2 SECOND‐HAND ACCOUNTS ON GALL IN VIENNA

### 2.1 Sniadecki

We begin with an account by Andrew (or Jędrzej) Sniadecki (1768–1838), not because he was the first to publish his account, but because he learned about Gall's system very early in its development. This author was born in Żnin, in the Polish–Lithuanian Commonwealth (Sakalauskaitė‐Juodeikienė, Eling, & Finger, 2017). After completing his medical studies in Vilnius, he went to Vienna, where he worked in hospitals from 1795 to 96 and became acquainted with Gall and his ideas. He then returned to his homeland to become Professor of Natural Sciences at Vilnius University.

Once back in Vilnius, Sniadecki informed his students, fellow physicians, and others about Gall's new science. Importantly, he published an article in 1805 that he titled, *Krótki Wykład Systematu Galla z przyłączniem niektórych uwag nad iego Nauką* [Short Lecture on the System of Gall with some Comments about his Science]. His informative piece appeared in the journal *Dziennik Wilenski* (see Sakalauskaitė‐Juodeikienė et al., 2017). In this article, he mentioned that Gall had discussed two organs devoted to life itself: an organ associated with a vital force and an organ of binding (tenacity) to life. He stated that Gall situated the brain tissue he associated with the life force in the posterior medulla oblongata, near the upper end of the spinal cord, and the organ for binding to life near the foramen magnum, while also mentioning the corpus callosum, then meaning not the great cerebral commissure but underlying white matter.

### 2.2 Bojanus

Ludwig Heinrich Bojanus (1776–1827), second in this series, was also affiliated with the University of Vilnius. He was born in Bousville (Buchsweiler), Alsace, and studied medicine at the University of Jena, graduating in 1797. He then traveled to Vienna, where he practiced in the General Hospital from 1797 to 98, shortly after Sniadecki had worked there. Also like Sniadecki, he attended some of the lectures Gall gave at his house, taking copious notes. He published them in 1802, 3 years prior to Sniadecki's (1805), although his fellow countryman had listened to Gall lecture before him (Bojanus, 1801, 1802 also see Sakalauskaitė‐Juodeikienė et al., 2017; and Sakalauskaitė‐Juodeikiene, Eling, & Finger, in press).

Bojanus started by relating how Gall contended that the medulla oblongata is the seat of the organ responsible for Tenacity of Life, since “there are no speedier means of killing an animal than to cut the medulla oblongata” (Bojanus, 1802, p. 81). He further mentioned that Gall had other noncortical faculties. In particular, he associated an area “a little further forward in the medulla oblongata,” where the medulla enters the skull with the Organ of the Love of Life or of the Instinct of Self‐Preservation. Bojanus indicated, however, that Gall was somewhat unsure about this faculty and was still evaluating the evidence for it (p. 81). One type of evidence he considered to be especially relevant for it came from cases of suicide. Such instances cannot be found in animals, only in humans, and Gall informed his audience that he had observed pathologies in this medullary area in cases of suicide.

Bojanus then mentioned a third faculty and its material organ, one Gall called the Organ for Choice of Nourishment. He localized it in the quadrigemini tubercles (colliculi), reasoning that the anterior tubercles are larger in carnivores, that the posterior tubercles are bigger in graminivores (literally “grass‐eating” or herbivorous animals), and that these structures are of equal size in omnivores.

### 2.3 Froriep

Having finished his basic medical education in Jena, Ludwig Friedrich Froriep (1779–1847) went on a study trip in 1799. It allowed him to meet and listen to Gall in Vienna, and he provided a detailed account of where Gall's faculties stood at this moment in time in his 1802 book, *Darstellung der ganzen, auf Untersuchungen der Verrichtungen des Gehirns gegründeten, Theorie der Physiognomik des Dr. Gall in Wien,* which translates as “Account of the Entire Theory of Physiognomy of Dr. Gall in Vienna Based on Investigations of the Brain” (Froriep, 1802). It followed a shorter piece that Froriep (1801) had just written on Gall.

In his book, Froriep followed Gall faithfully by first discussing his guiding principles. He then described his faculties under the heading *Stufenleiter der Veredlung der Thiere* [Ladder of Ennoblement of Animals], Gall's version of the *scala naturae* or Great Chain of Being. His first step involving animals included creatures like polyps, which lack real nervous systems and remain stationary. At the second stage, one finds animals with a spine and primitive brain—animals that are capable of moving and sensing. Here Gall brought up worms, which will die if the spine is cut off from the brain, with Froriep explaining:

If one lesions such an animal, not all parts remain alive equally long, but only that part seems to remain alive, which is the upper end part of the nerves [*Streifen*] that constitute the spinal cord of the animal. Gall thinks he is correct in assuming that the vital force [*Organ der Lebenskraft*] is located in a place that can be seen in all more complete animals … namely in the oblong medulla. (p. 43).

Froriep added that it is well known that destroying this part in “more complete animals,” as will happen, for instance, when one kills an ox, results in immediate death. He stated that Gall also held that the size of the hole (foramen magnum) where the spinal cord enters the skull is related to the tenaciousness of life. This is similar to what we found in Bojanus, who also mentioned the association between the width of the foramen magnum and the tenacity for life.

But unlike Bojanus, Froriep did not go on to include a faculty for food choices in the midbrain. Instead, he continued onto the third step of Gall's ladder, where he placed animals that propagate via copulation. “In all animals that use this method for propagation,” he related, “one finds above the upper end of the spinal cord—that is, beyond the organ of vital force—two little knots that contain the organ of propagation (*Organ des Begattungstriebes*),” which in higher animals develops into the cerebellum (p. 44; see also Eling & Finger, 2019).

Thus, more than any of the preceding authors, Froriep presented Gall's faculties and organs, including his noncortical faculties and their organs, within an evolutionary framework, something Gall was doing and would continue to do in his later books.

### 2.4 Walther

Philipp Franz Walther (1782–1849), born in the small German village of Burrweiler some 30 miles (50 km) northwest of Karlsruhe, studied medicine in Vienna and obtained his medical doctorate from the University of Landshut in 1803. During the year preceding his graduation, he published his *Critische Darstellung der Gall'schen anatomisch‐physiologischen Untersuchungen des Gehirn‐und Schädel‐baues* [Critical Account of Gall's Anatomical‐Physiological Investigations of the Brain and the Form of the Skull] (Walther, 1802).

Just before Walther began to describe Gall's various organs (on p. 77), he, like Froriep, related how the nervous systems of different animals permits them to interact with their environments. He then began his overview of the faculties, beginning with the *Organ der Lebenskraft*, the organ of a vital force, which Gall continued to localize in the oblong medulla. Walther did not think this was the best name for this organ or its presumed function, although he could not come forth with a good alternative, further noting that Gall “wants to declare with this expression the indicated area of the brain as the center of the sensitive system.” Walther also mentioned how the faculty for the vital force related to the width of the magnum foramen. He then continued on to how medullary tissue relates to the cerebellum, where Gall was locating the sexual drive (*Geslachtstrieb*).

## 3 THIRD‐HAND ACCOUNTS FROM VIENNA

### 3.1 Villers

Gall's views were also presented and discussed by authors who had not heard him directly, but had read what others wrote about him and, orally or through letters, had discussions with people knowledgeable about his doctrine. Charles de Villers (1765–1815), who was born in Boulay‐Moselle in the Lorraine region, fled from the French Revolution and settled in Lübeck, in the Danish‐German State of Schleswig‐Holstein, was one such person. In 1802, he sent a lengthy letter to comparative anatomist Georges Cuvier (1769–1832), Permanent Secretary of the *Académie des Sciences* in Paris. It was titled, *Lettre à G. Cuvier sur une Nouvelle Théorie du Cerveau par Gall: ce Viscère Étant Considéré comme l’Organe Immédiat des Facultés Morales* [Letter on a New Brain Theory by Doctor Gall: This Viscera Being Considered as the Immediate Organ of the Moral Faculties].

Villers had read Gall's (1798) letter to Retzer and Froriep's (spelled Froriess in his *lettre*) account. He had also talked with physician Georg Heinrich Behn (1773–1855), who obtained his medical degree from Jena in 1796 and had then gone on a study tour to several cities, including Vienna, where he stayed for 10 months and met Gall. Behn was now a neighbor in Lübeck, the city where he was born. Villers did not meet with Gall or attend his lectures. He relied solely on other sources.

Having dealt with the more fundamental assumptions underlying Gall's theory, Villers listed Gall's faculties in the second half of his letter, mentioning only 22. He began with the *Organe de la force vitale* [Organ of vital force], situated just above the spinal cord in the medulla oblongata. The larger the foramen magnum is, he related, the more vital force there will be. His second organ was for *la force génératrice*, the organ for propagation, which Gall located in the cerebellum.

### 3.2 Leune

Johann Carl Friedrich Leune (1757–1825) also did not meet Gall. Leune studied medicine in Leipzig and lectured physiology, pathology and ophthalmology at that city's university, and had been invited by his publisher to comment on Villers’ (1802) *Lettre*. Rather than writing a short reply, he penned his *Entwickelung der Gall'schen Theorie über das Gehirn* [Development of Gall's Theory of the Brain], published in 1803. At the end of his introduction, he indicated that, in addition to reading Villers’ report to Cuvier, he had also consulted Froriep's and Walther's books. He did not mention these authors’ names, only their book titles.

As in most other accounts, Leune first addressed Gall's philosophical premises, in particular, the question whether he should be branded a materialist. He then turned to his individual organs, starting with his organ for a vital force. Leune's description of this organ closely resembles Villers’. His second organ was for propagation, and here too what he wrote followed Villers’ description.

### 3.3 Vrolik

Dutch anatomist Gerardus Vrolik (1775–1859), who taught at Amsterdam's Athenaeum Illustre, published a book titled *Het Leerstelsel van Joseph Gall* [The Doctrine of Joseph Gall] in 1804 (Vrolik, 1804). This was 2 years after Villers’ famous *Lettre*, a year after Leune's book came out, and just a year before Gall left Austria to lecture in various German cities and elsewhere. Vrolik followed Villers and Leune in drawing on information provided by others. His major source was, in fact, Leune (1803). A Dutch translation of Leune's book had been prepared by Martinus Stuart (1765–1826), to whom we shall return below. He apparently also consulted the works by Bojanus and Walther. Hence, his rendition, being based on second‐ and third‐hand accounts, can be regarded as an even more distant fourth‐hand account.

Vrolik began his 1804 volume with a short, general introduction, placing Gall's doctrine in the tradition of theories relating the mind to the brain. In this same context, he mentioned Petrus Camper (1722–1798), Samuel Thomas von Soemmerring (1755–1830), Georg Ernst Stahl (1659–1734), and others. He also provided a short introduction to Gall's system, mentioning three kinds of faculties: those responsible for sustaining and propagating a species, those governing “all sorts of tendencies and passions,” and intellectual faculties.

Vrolik stated that Gall had identified 30 faculties of mind. Writing in Dutch, he called the organ for Gall's first faculty *Het werktuig of de kracht des levens* [The organ of the force of life]. He related that not all animals are equally equipped to oppose forces that undermine life, and mentioned that Gall was quite certain that this organ could be localized in the oblong medulla. In addition, he wrote that Gall thought the width of the medulla could explain why women can sustain life better than men. He then added to what Gall had been stating with some of his own observations on differences in foramen magnums in different groups of people, including non‐Europeans.

The second faculty on Vrolik's list was the one associated with the Organ of Self‐Preservation. Vrolik, like Bojanus, mentioned how Gall felt that suicide cases were particularly relevant for understanding this faculty and the location of its material organ. He maintained, however, that Gall still seemed uncertain about the organ's precise locus, although he had opined that it must be located at a higher level than the primitive medullary region situated just above the spinal cord.

Gall's third organ, Vrolik continued, determines an organism's choice of food. It can account for distinctions between herbivorous, carnivorous, and omnivorous animals, and is located in the anterior and posterior tubercles. Here too, Vrolik's representation closely resembles what others, including Bojanus 2 years earlier, had written.

### 3.4 Doornik

Dutch physician Jacob Elisa Doornik (1777–1837), Vrolik's colleague at the Athenaeum Illustre, published a book on Gall and his doctrine at the same time as Vrolik did (Doornik, 1804). It bore the title *De Herssen Schedelleer van Frans Joseph Gall getoetst aan de Natuurkunde en wijsbegeerte* [Franz Joseph Gall's Theory on the Brain and the Cranium, Tested by Physics and Philosophy]. Doornik added his own drawing of a skull showing the locations of Gall's organs. Like Vrolik, Doornik had not attended Gall's lectures when he wrote this book, relying heavily on Walther and Leune for his presentation of Gall's *Schädellehre*.

Doornik's introduction is much more comprehensive than Vrolik's. Still, in accord with his predecessors’ accounts, he too opted to begin with the fundamental assumptions underlying organology, which seemed to be how Gall was starting his lectures. The third section of his book is most pertinent here, because this is where he followed Gall in presenting the brain as an “aggregation” of organs for different mental capacities, propensities, and impressions that can be discerned from the skull. Doornik discussed each of Gall's organs, adding his own critical remarks.

Doornik, like his countryman Vrolik, tried to add more structure to Gall's rapidly‐changing list of faculties. He called his first section “Organs for the Preservation and Propagation of the Existence,” and he included four faculties under this heading. He began with the Organ of Vital Force, briefly summarizing Gall's arguments for it. He localized it in the medulla oblongata, also mentioning the size of the foramen magnum.

Gall's second faculty, mentioned in a way that resembles Froriep's description, was the one that had to do with sexual drive. Once again, it is associated with two little knots at the upper end of the spinal cord in animals with primitive brains, and with the cerebellar hemispheres in those higher on what Gall considered the organizational ladder.

## 4 DETAILED REPORTS BY OTHERS FROM GALL'S LECTURE TOUR

### 4.1 Bischoff

Christian Heinrich Ernst Bischoff (1781–1861), born in Hanover, obtained his medical degree in Jena, in 1801. In 1804, he was appointed Extraordinary Professor of Physiology in Berlin. There, early in 1805, Bischoff attended the first series of lectures Gall gave on his lengthy scientific tour (Bischoff, 1805). Since Gall had not published anything about his organology since his 1798 public letter to Viennese censor Joseph Friedrich Freiherr von Retzer (1754–1824), Bischoff responded to the need for an informative book about Gall and where his revolutionary doctrine now stood.

Bischoff called his secondary account *Darstellung der Gall'schen Gehirn‐und Schädel‐Lehre* [Presentation of Gall's Brain and Skull Theory]. He began by summarizing Gall's doctrine, and then worked through his faculties, providing information about each function and its dependence on a specific organ in the brain, particularly as revealed by skull markers in unusual and extraordinary people (criminals, the insane, great musicians, etc.) and a myriad of animals.

His treatise shows that Gall was no longer lecturing about brainstem organs for a vital force, tenacity to life, or choice of nourishment. These items had apparently been removed from Gall's list just before he left Vienna or when making his way to Berlin. Hence, his list started with Gall's *Organ der Geschlechtsliebe*—which would be called “The Organ of Sexual Love” in an 1807 English translation of Bischoff's (1807) book. Called the reproductive instinct or drive by others, it's organ was still in the cerebellum. It would henceforth be Gall's most primitive faculty of mind, and the only faculty on his final list with a non‐cerebral material organ (see Sakalauskaitė‐Juodeikienė, Eling, & Finger, in press).

### 4.2 Stuart

We now turn to Martinus Stuart, mentioned above, for our final account about where Gall's system of faculties and organs stood before he entered France. Stuart was Dutch, but unlike his countrymen Vrolik and Doornik, what he conveyed was based on attending Gall's lectures, which he did after Gall crossed the border and began speaking in the Netherlands. Also setting him apart from the other authors mentioned here, Stuart was not trained as a physician. He had studied theology and served as a minister, and he had established himself as an esteemed historian.

Stuart set forth to describe each of the 10 lectures Gall delivered in Amsterdam in 1806, the year before he entered France. He called his contribution *Herinneringen uit de lessen van Frans Joseph Gall* [Memories from Franz Joseph Gall's Lessons]. It quickly went to press, appearing during the same year as Gall's visit.

Stuart (1806) informed his readers that Gall was now listing 27 organs of mind. Another noteworthy feature of his rendition in the present context is that he wrote that Gall had stated that large lesions of the oblong medulla lead to immediate death. Although this was something noted in some of the other earlier accounts, Stuart went on to add that Gall was now claiming that this fact should not be considered as proof of a *special* (our emphasis) organ for *Levenskracht*, the vital force. On the contrary, Gall was maintaining that he had never accepted such a force, although many others had written that he had previously made this assertion!

Stuart went on to clarify Gall's position—in effect, that there is no reason to consider the vital force as a metaphysical entity. Further, the medulla oblongata merely serves as the connection between organic (*werktuiglijk*) life and animal life. Hence, removing this connection ends “life‐consciousness,” including voluntary control over body parts. This, he emphasized, is not the same as destroying an organ associated with a faculty of mind.

This said, Stuart's list, like Bischoff's, began with the drive for reproduction. He used the term *Geslachtsdrift* (sexual drive) for it. And, as in all other known accounts to this point in time, he informed his readers that Gall located its material organ in the cerebellum.

## 5 DISCUSSION

When referring to Gall's faculties and organs of the mind, modern authors have routinely discussed only his final grouping of 26 cerebral cortical organs, of which he classified eight as distinctly human, and, appearing first on his ascending list, his only cerebellar organ, the one associated with a higher‐order reproductive drive. Rarely mentioned is how Gall's list of faculties, from most primitive to most exalted, changed before he began publishing his final list in his “great work” and its less‐expensive follow‐up edition (Gall & Spurzheim, 1810–19; Gall, 1822–25, 1835[Link]).

Looking at different renditions of Gall's theory, starting while he was still in Vienna and ending before he settled in France in 1807, shows how he was actively pondering what would remain or be added to his list of faculties and, in some instances, the locations of the organs necessary for specific faculties to function in everyday life. Particularly notable in this context, and the focus of this contribution, is how Gall at one time had medullary and midbrain organs that would not survive the cut, in addition to his cerebellar organ for reproduction, which he would defend to his dying day (Eling & Finger, 2019).

For a better understanding of what seemed to be going through Gall's mind as he was attempting to finalize his list of faculties and the loci of their material organs, it is necessary to stress that we could do no more than examine the accounts of a select group of individuals familiar with Gall's ideas. All of these men were learned, all but one (i.e., Stuart) had been a physician, and many (but by no means all) had attended his lectures, giving added credence to their reports, which served as the basis for some third‐ and fourth‐hand accounts. In particular, the reports from Froriep (1802), Walther (1802), and Bojanus (1802) proved very influential in guiding what other would write about Gall's doctrine before Gall finally started to publish his long‐awaited books in 1810.

It bears repeating that Gall had not published an article, a pamphlet, or a book listing his faculties and organs, along with the evidence for each one, before 1810. Among the reasons he gave for delaying his “great work” were that he needed more time to collect additional evidence for his assertions and that he wanted additional feedback to be able to silence his critics with what he would include in the volumes themselves. Hence, some of the finer details in the early reports by others could reflect what his listeners thought he was saying, as contrasted with what he was actually trying to present to his audiences. Moreover, some renditions might have been “colored” by the personal biases of his listeners and those “borrowing” from preceding reports. While some of these people might have been open‐minded, others, Villers being a prime example, might have been influenced by religion, time‐honored notions, ambition, and the politics of the day.

### 5.1 Questions surrounding the vital force

Several early authors, as we have seen, maintained that Gall had presented the vital force as his most primitive faculty, the one he associated with a region of the lower medulla oblongata, just above the spinal cord. The fact that this organ kept appearing in the earlier lists might suggest that these early accounts should be regarded as trustworthy. Nonetheless, there is some history to consider here along with considerable murkiness.

Many German‐speaking *Naturphilosophen,* but also others at this time, were associating a vital force—one independent of known physical and chemical forces—with the evolution and development of living organisms. These philosophers, now labelled “vitalists,” envisioned the vital force as *the* causative factor underlying the development and organization of the body parts, as well as life and death. Gall, however, was by no means a vitalist, raising the possibility that what he was actually saying about the faculty and *Organ des Lebenskraft* could have been misinterpreted.

There are several reasons for entertaining this possibility. One is that the ancient notion of an invisible life force was becoming less acceptable during the Age of Enlightenment, despite the efforts of German *philosoph* Georg Ernst Stahl (1660–1734) and others to revive or keep the metaphysical concept alive. Gall considered himself a forward‐looking, enlightened scientist, not tied to the past. Second, in his book on medicine published in 1791, 5 years before he even began to lecture on his more famous, skull‐based doctrine, he relentlessly attacked Stahl and all other metaphysicians as backward and misguided in how they perceived the body when healthy and when diseased (Gall, 1791). Third, Gall made it clear in all of his later writings that he favored an empirical approach to science and medicine, an orientation that meant discarding everything that smacked of metaphysics (e.g., souls, vital forces, etc.) in favor of what could be experienced through the senses (vision, touch, etc.). And fourth, Stuart had written in his 1806 account that Gall had contended that he never listed the vital force as a faculty of mind.

From this time on, the vital force would not appear on the lists provided by others of Gall's special faculties. Nor would Gall list it in his own books. Although not in this way, he would, however, bring up the notion of a vital force or principle in his volumes. In a paragraph titled “Influence of Wants on the Instincts, Propensities, and Faculties of Animals, and of Man,” for example, he would write:

The reader will now be convinced, that there cannot exist any necessity or natural occasion, without there existing an active organ, an impulse from within. Without certain vital forces in the interior, there could be neither hunger nor thirst, nor necessity for respiration, nor necessity of the union of the sexes. Thus the exterior necessities always suppose an interior force. (Gall, 1835[Link], 18, vol. 1, pp. 154–155).

It is important to emphasize that, when alluding to “vital forces in the interior,” Gall was not falling back on a metaphysical construct, like some sort of a guiding soul. He was merely trying to indicate that an organ could become physiologically active, not how this might be accomplished. His nonmetaphysical use of the term would be made even clearer when he would write: “In all my research the question was to discover, not the vital functions or the reciprocal vital influences of the different parts of the nervous system, but the animal functions, moral qualities, and intellectual faculties, and the seats of their organs” (Gall, 1835[Link], vol. 6, p. 173).

Still another quote helps us to understand even more what Gall was trying to state about vital properties or forces:

Cuvier distinguishes very correctly, the vital properties of the brain from their particular functions. “We shall see, hereafter, whether the function, that M. Flourens believes may be attributed to the cerebellum, is not rather a special function, than a vital property common to the medulla spinalis and the medulla oblongata.” We must first know the special functions; that is to say, those which constitute animal life, before we can obtain a knowledge of them by means of ablation … But up to the present time, these special functions have not been known (Gall, 1835[Link], vol. 6, p 243–244).

This quotation was taken from Gall's critique of Jean Pierre Marie Flourens’ (1794–1867) cerebellar lesion experiments on birds (discussed in Eling & Finger, 2019; Finger & Eling, 2019). It further illustrates how Gall distinguished between the vital or activating features and the special features of a faculty, and how his focus was on special animal functions, and not imperceptible, mysterious forces that seemed beyond his grasp and were speculative given the limitations of the senses and the tools of his day.

### 5.2 Resurrecting some noncortical faculties

Let us now turn to Gall's two other noncortical faculties, namely tenacity for life and choice of nourishment. These faculties, along with reproduction (cerebellum), caring for offspring (posterior cerebral cortex), and others, reflect how Gall was attempting to explain specific propensities, instincts, and basic behaviors contextually. Thus, while others were classifying certain acts as vegetative, instinctual, or automatic behaviors, Gall was framing these same activities as ways of successfully interacting with the environment and assuring the very survival of the species.

Importantly, Gall associated these functions with the mind in higher organisms. As he framed it, they involve consciousness and making choices, and therefore are “intellectual” processes. This realization helps explain why Gall chose to integrate some of what he had been stating about his subcortical faculties with the faculties having cortical organs on his final list. In reality, these subcortical faculties were not completely discarded, but instead were integrated into what he would write about higher functions and how they depended on cortically based organs.

Consider Tenacity for Life, a faculty mentioned by Bojanus and Vrolik, and one that Gall had initially associated with the medulla oblongata. In his later works, Gall would write that “Snails, lobsters, and lizards, not only endure the most severe wounds, but reproduce, even several times, parts that they have lost, such as the feet, eyes, and head,” and that “Tenacity of life diminishes in proportion as the brain becomes more complex” (Gall, 1835[Link], vol. 2, p. 40).

To be clear, Gall did not directly employ his earlier notion of the tenacity of life in his descriptions of his 27 distinct cortical organs. But he did continue to devote considerable attention to cases of suicide, especially when describing “Carnivorous Instinct; Disposition to Murder” (Gall, 1835[Link], vol. 4). This is especially notable in a paragraph on “The Influence of the Brain on the Cranium in Subjects Predisposed to Suicide” (vol. 3, p. 62), and in other places in his two sets of volumes in French and the English translation of his *Sur les Fonctions du Cerveau et sur Celles de Chacune de ses Parties*, where he discussed suicide and the brain (e.g., Gall, 1835[Link], vol. 4, pp. 96, 98, 100).

Also consider the fate of his faculty for choice of nourishment; the fact that some kinds of animals are herbivorous while others eat flesh and so forth. Early on, he associated this faculty with the tubercles, or colliculi in modern parlance. In Gall's final list of faculties, choice of nourishment is incorporated into his fifth faculty “Carnivorous Instinct; Disposition to Murder.” Here he informs his readers that murder is not always an immoral or criminal act, and that some animals have a tendency to kill to feed and survive, whereas others do not.

I here confine myself exclusively to the carnivora, properly so called. Although they are all destined to devour other animals, there exist great differences among them relative to their murderous instinct. Many of them kill only such as are necessary for their subsistence; while others, like the weasel, tiger, etc., without impelled by hunger, kill and tear in pieces every living creature around them. The difference between dogs in this respect, proves to demonstration, that hunger and thirst for blood are not the sole motives that determine animals to slay one another. … All dogs are carnivorous; they prefer flesh to any other nourishment; still there are some in which we can hardly observe the carnivorous instinct, and which, surrounded by birds, mice, and hares, manifest no wish to destroy them. (Gall, 1835[Link], vol. 4, p. 55).

### 5.3 In retrospect

Gall's faculties and their associated organs were very much in flux before he arrived in Paris in 1807. Although we did not opt to go into how he renamed some of his cortical faculties or wrestled with the locations of their material organs, this survey of early reports reveals how he seemingly included three noncortical faculties on some of his early lists. As also noted, he later claimed that he never included a special faculty for a vital force on his lists, reframed what he had written about “tenacity for life,” and moved some of what he had to say about a faculty involved with food choices into a cortical faculty of mind (Carnivorous Instinct; Disposition to Murder) on his final list.

Looking at what Gall was telling his audiences about the faculties and their organs through his doctrine's first decade shows that, although he would establish a reputation for being very dogmatic and brutal to his critics, his own mind was, in fact, open to change while he was lecturing in Vienna. He continued making changes in his list of faculties and the locations of their organs as he travelled through many European cities, collecting additional evidence for each and thinking deeply about the brain and his doctrine, before making Paris his new home.

How Gall struggled to finalize his list of faculties and their loci has for too long been an overlooked part of the history of what Gall was calling his *Schädellehre* (doctrine of the skull) in German and *organologie* in French. As with many other revolutionary ideas, what first emerged in a founder's fertile mind would undergo changes. Gall understood that his early lists needed to be refined, and how he began with several noncortical organs and ended with none was an important step in the development and emergence of one of the most important and influential scientific fads of the 19th century—his so‐called new science that would become widely known as “phrenology.”
